# Supplementary figures and images for: Effect of motivated physicians and elderly patients with hypertension or type 2 diabetes mellitus in prepared communities on health behaviours and outcomes: A population-based PS matched retrospective cohort study during five-year follow-up period
Source: PLoS One. 2024 Feb 13;19(2):e0296834. doi: 10.1371/journal.pone.0296834 (PMC10863870; doi:10.1371/journal.pone.0296834)

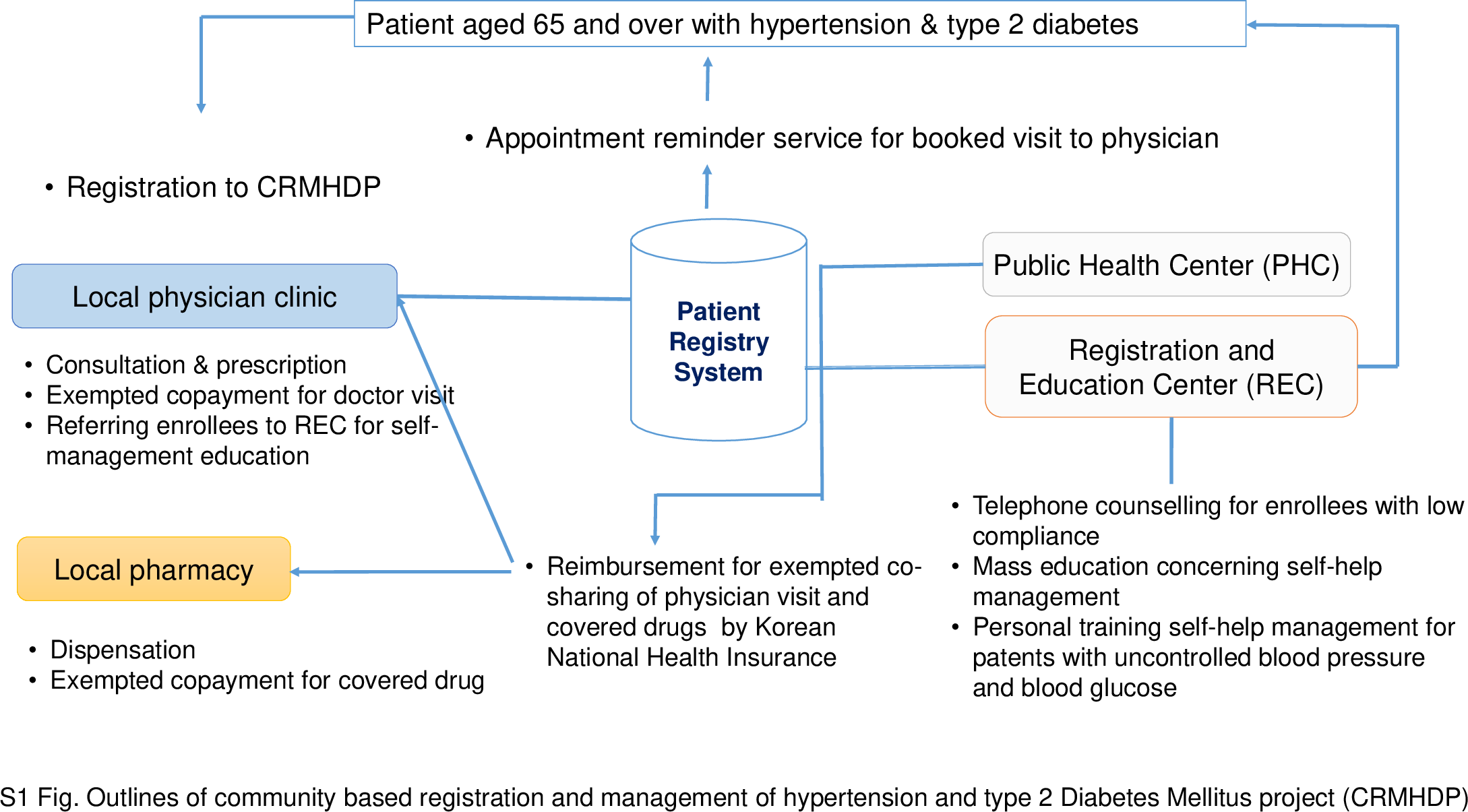

Supplement: S1 Fig — (TIF) [file pone.0296834.s001.tif]

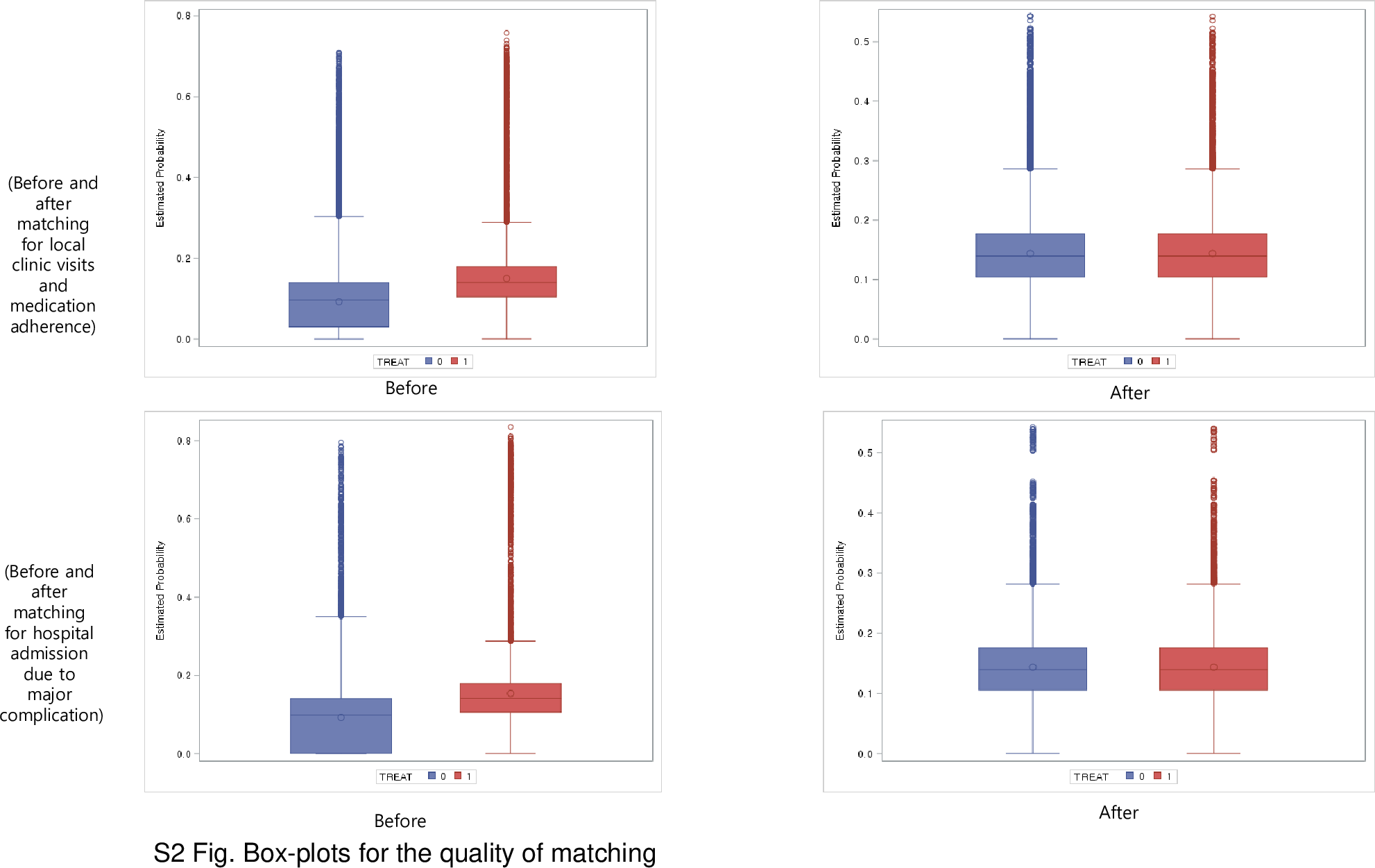

Supplement: S2 Fig — (TIF) [file pone.0296834.s002.tif]
