# Supplementary material for: Effect of motivated physicians and elderly patients with hypertension or type 2 diabetes mellitus in prepared communities on health behaviours and outcomes: A population-based PS matched retrospective cohort study during five-year follow-up period
Source: PLoS One. 2024 Feb 13;19(2):e0296834. doi: 10.1371/journal.pone.0296834 (PMC10863870; doi:10.1371/journal.pone.0296834)
Supplement: S1 Table — (PDF) [file pone.0296834.s003.pdf]

S1 Table. Balance in measured baseline variables before and after matching (included prior to any complications existing).

Variables	Before matching					After matching				
	Intervention	Control	t/x²	P-value	D	Intervention	Control	Z	P-value	D
**Total**	48,325	442,199				46,865	93,730			
**Sex: no, (%)**			49.24***	<.0001	0.033			0.04	0.9705	0.000
**Male**	17,753 (36.74)	169,673 (38.37)				17,202 (36.71)	34,395 (36.70)			
**Female**	30,572 (63.26)	272,526 (61.63)				29,663 (63.29)	59,335 (63.30)			
**Age: mean, ± SD***			-18.24***	<.0001	0.087			-1.64	0.1011	0.008
**65–69**	66.84 ± 1.47	66.57 ± 1.47				66.84 ± 1.47	66.56 ± 1.48			
**70–74**	71.90 ± 1.40	71.88 ± 1.41				71.90 ± 1.40	71.90 ± 1.41			
**75–79**	76.74 ± 1.39	76.75 ± 1.40				76.74 ± 1.39	76.77 ± 1.40			
**80–84**	81.71 ± 1.39	81.65 ± 1.38				81.71 ± 1.38	81.64 ± 1.38			
**85–89**	86.67 ± 1.31	86.58 ± 1.32				86.67 ± 1.31	86.58 ± 1.32			
**90 +**	92.12 ± 2.30	92.12 ± 2.48				92.16 ± 2.33	92.20 ± 2.52			
**Income quantile: no, (%)**			602.72***	<.0001	0.055			0.54	0.5868	0.004
**1st quantile (poorest)**	9,679 (20.03)	78,987 (17.86)				9,346 (19.94)	18,546 (19.79)			
**2nd quantile**	6,174(12.78)	52,752(11.93)				5,946 (12.69)	11,920 (12.72)			
**3rd quantile**	7,538 (15.60)	61,461 (13.90)				7,296 (15.57)	14,644 (15.62)			
**4th quantile**	9,631 (19.93)	84,640 (19.14)				9,340 (19.93)	18,662 (19.91)			
**5th quantile**	15,303 (31.67)	164,359 (37.17)				14,937 (31.87)	29,958 (31.96)			
**Findings or coexisting conditions at admission: no, (%)**			248.49***	<.0001	0.047			1.31	0.1898	0.1898
**History of hypertension**	32,633 (67.53)	288,833 (65.32)				31,661 (67.56)	63,009 (67.22)			
**History of type 2 diabetes mellitus**	4,764 (9.86)	39,361 (8.90)				4,629 (9.88)	9,244 (9.86)			
**History of hypertension and type 2 diabetes mellitus**	10,928 (22.61)	114,005 (25.78)				10,575 (22.56)	21,477 (22.91)			
**Type of physician speciality: no, (%)**			20689***	<.0001	0.520			3.11**	0.0019	0.015
**Internal medicine and family medicine**	41,026 (84.90)	277,524 (62.76)				40,367 (86.13)	81,222 (86.66)			
**Others**	7,299 (15.10)	164,675 (37.24)				6,498 (13.87)	12,508 (13.34)			
**Type of public health insurance: no, (%)**			140.55***	<.0001	0.056			0.36	0.7163	0.001
**National Health Insurance (self-employed)**	17,054 (35.29)	144,314 (32.64)				16,480 (35.16)	32,890 (35.09)			
**National Health Insurance (employees)**	31,099 (64.35)	296,078 (66.96)				30,218 (64.48)	60,479 (64.52)			
**Medical aid**	172 (0.36)	1,807 (0.41)				167 (0.36)	361(0.39)			
**PDC[1] > 1 yr: mean, ± SD**			-78.30***	<.0001	0.372			0.68	0.4969	0.002
**< 290<br>> = 290**	213.0 ± 123.3	166.7 ± 125.6				208.6 ± 128.6	208.9 ± 137.1			
**PDC[1] > 2 yr: mean, ± SD**			-76.61***	<.0001	0.371			-1.84	0.0659	0.007
**< 290<br>> = 290**	200.5 ± 129.7	152.9 ± 127.0				196.7 ± 138.3	195.8 ± 137.9			

***$p < 0.001$, **$p < 0.05$, *$p < 0.1$.

Plus–minus values are means ± SD.
t/x^2 and Z indicate the test statistics obtained from independent t-test/Pearson Chi^2 and GEE, respectively.
CRMHDP: Community-based Registration and Management for Hypertension and Type 2 Diabetes mellitus Project.

[1]PDC (proportion of days covered) calculation will be the number of total days covered for drugs or injections divided by the number of total days each period.
