# Supplementary material for: Effect of motivated physicians and elderly patients with hypertension or type 2 diabetes mellitus in prepared communities on health behaviours and outcomes: A population-based PS matched retrospective cohort study during five-year follow-up period
Source: PLoS One. 2024 Feb 13;19(2):e0296834. doi: 10.1371/journal.pone.0296834 (PMC10863870; doi:10.1371/journal.pone.0296834)
Supplement: S2 Table — (PDF) [file pone.0296834.s004.pdf]

S2 Table. Balance in the measured baseline variables before and after matching (excluded prior to any complications existing)

Variables	Before matching					After matching				
	Intervention	Control	t/x²	P-value	D	Intervention	Control	Z	P-value	D
**Total**	27,936	255,276				27,242	54,484			
**Sex: no, (%)**			21.92***	<.0001	0.029			-0.96	0.3354	0.007
**Male**	10,422 (37.31)	98,902 (38.74)				10,165 (37.31)	20,507 (37.64)			
**Female**	17,514 (62.69)	156,374 (61.26)				17,077 (62.69)	33,977 (62.36)			
**Age: mean, ± SD**			-13.89***	<.0001	0.088			-0.86	0.3913	0.006
**65–69**	66.81 ± 1.46	66.53 ± 1.47				66.81 ± 1.46	66.53 ± 1.47			
**70–74**	71.86 ± 1.39	71.85 ±1.41				71.86 ± 1.40	71.87 ± 1.41			
**75–79**	76.71 ± 1.39	76.74 ± 1.40				76.71 ± 1.39	76.73 ± 1.40			
**80–84**	81.71 ± 1.38	81.65 ± 1.38				81.71 ± 1.38	81.67 ± 1.39			
**85–89**	86.72 ± 1.33	86.61 ± 1.34				86.74 ± 1.34	86.61 ± 1.34			
**90 +**	92.11 ± 2.21	92.20 ± 2.49				92.12 ± 2.23	92.39 ± 2.68			
**Income quantile: no, (%)**			319.66***	<.0001	0.049			1.58	0.1152	0.011
**1st quantile (poorest)**	5,683 (20.34)	46,975 (18.40)				5,522 (20.27)	10,800 (19.82)			
**2nd quantile**	3,621 (12.96)	31,246 (12.24)				3,508 (12.88)	7,115 (13.06)			
**3rd quantile**	4,372 (15.65)	35,789 (14.02)				4,240 (15.56)	8,466 (15.54)			
**4th quantile**	5,613 (20.09)	48,734 (19.09)				5,495 (20.17)	10,870 (19.95)			
**5th quantile**	8,647 (30.95)	92,532 (36.25)				8,477 (31.12)	17,233 (31.63)			
**Findings or coexisting conditions at reggisration: no, (%)**			30.19***	<.0001	0.029			1.21	0.2264	0.017
**History of hypertension**	18,512 (66.27)	165,663 (64.90)				18,078 (66.36)	35,712 (65.55)			
**History of type 2 diabetes mellitus**	2,694 (9.64)	24,271 (9.51)				2,621 (9.62)	5,351 (9.82)			
**History of hypertension and type 2 diabetes mellitus**	6,730 (24.09)	65,342 (25.60)				6,543 (24.02)	13,421 (24.63)			
**Type of physician speciality: no, (%)**			13,261***	<.0001	0.548			-0.57	0.5680	0.003
**Internal medicine and family medicine**	23,933 (85.67)	159,567 (62.51)				23,805 (87.38)	47,668 (87.49)			
**Others**	4,003 (14.33)	95,709 (37.49)				3,437 (12.62)	6,816 (12.51)			
**Type of public health insurance: no, (%)**			79.20***	<.0001	0.053			0.78	0.4357	0.005
**National Health Insurance (self-employed)**	9,778 (35.00)	82,941 (32.49)				9,500 (34.87)	18,863 (34.62)			
**National Health Insurance (employees)**	18,090 (64.76)	171,430 (67.15)				17,675 (64.88)	35,481 (65.12)			
**Medical aid**	68 (0.24)	905 (0.35)				67 (0.25)	140 (0.26)			
**PDC[1] > 1yr: mean, ± SD**			-58.06***	<.0001	0.367			3.28**	0.0010	0.017
**< 290<br>> = 290**	191.1 ± 127.3	144.6 ± 125.8				203.8 ± 131.3	206.1 ± 139.9			
**PDC[1] > 2yr: mean, ± SD**			-53.67***	<.0001	0.345			0.87	0.3853	0.004
**< 290<br>> = 290**	178.4 ± 132.1	133.9 ± 125.6				191.3 ± 141.2	191.8 ± 141.1			

***$p < 0.001$, **$p < 0.05$, *$p < 0.1$.

Plus–minus values are means ± SD.
t/x^2 and Z indicate the test statistics obtained from independent t-test/Pearson Chi^2 and GEE, respectively.
CRMHDP: Community-based Registration and Management for Hypertension and Type 2 Diabetes mellitus Project

[1]PDC (proportion of days covered) calculation will be the number of total days covered for drugs or injections divided by the number of total days in a given period.
