# Supplementary material for: Effect of motivated physicians and elderly patients with hypertension or type 2 diabetes mellitus in prepared communities on health behaviours and outcomes: A population-based PS matched retrospective cohort study during five-year follow-up period
Source: PLoS One. 2024 Feb 13;19(2):e0296834. doi: 10.1371/journal.pone.0296834 (PMC10863870; doi:10.1371/journal.pone.0296834)
Supplement: S3 Table — (PDF) [file pone.0296834.s005.pdf]

S3 Table. Baseline patient demographic and clinical characteristics of propensity score-matched intervention and control patients in the study (included prior to any complications existing).

Demographic Variables		Total		Intervention		Control	
		n	%	n	%	n	%
Total		140,595	100.00	46,865	100.00	93,730	100.00
Sex	Male	51,597	36.70	17,202	36.71	34,395	36.70
	Female	88,998	63.30	29,663	63.29	59,335	63.30
Age	65–69	55,344	39.36	18,103	38.63	37,241	39.73
	70–74	39,008	27.74	13,531	28.87	25,477	27.18
	75–79	25,126	17.87	8,523	18.19	16,603	17.71
	80–84	13,621	9.69	4,379	9.34	9,242	9.86
	85–89	5,865	4.17	1,839	3.92	4,026	4.30
	90 +	1,631	1.16	490	1.05	1,141	1.22
Income quantile	1st quantile (poorest)	27,892	19.84	9,346	19.94	18,546	19.79
	2nd quantile	17.866	12.71	5,946	12.69	11,920	12.72
	3rd quantile	21,940	15.61	7,296	15.57	14,644	15.62
	4th quantile	28,002	19.92	9,340	19.93	18,662	19.91
	5th quantile	44,895	31.93	14,937	31.87	29,958	31.96
Findings or co-existing conditions at admission	History of hypertension	94,670	67.34	31,661	67.56	63,009	67.22
	History of diabetes mellitus	13,873	9.87	4,629	9.88	9,244	9.86
	History of hypertension and diabetes mellitus	32,052	22.80	10,575	22.56	21,477	22.91
Type of physician speciality	Internal medicine and family medicine	121,589	86.48	40,367	86.13	81,222	86.66
	Others	19,006	13.52	6,498	13.87	12,508	13.34
Type of public health insurance	National Health Insurance (self-employed)	49,370	35.12	16,480	35.16	32,890	35.09
	National Health Insurance (employees)	90,697	64.51	30,218	64.48	60,479	64.52
	Medical aid	528	0.38	167	0.36	361	0.39
PDC[1] > 1yr	< 290	80,709	57.41	27,955	59.65	52,754	56.28
	> = 290	59,886	42.59	18,910	40.35	40,976	43.72
PDC[1] > 2yr	< 290	85,241	60.63	28,232	60.24	57,009	60.82
	> = 290	55,354	39.37	18,633	39.76	36,721	39.18

***$p < 0.001$, **$p < 0.05$, *$p < 0.1$.

[1]PDC (proportion of days covered) calculation represents the number of total days covered divided by the number of total days in each period.
