# Supplementary material for: Effect of motivated physicians and elderly patients with hypertension or type 2 diabetes mellitus in prepared communities on health behaviours and outcomes: A population-based PS matched retrospective cohort study during five-year follow-up period
Source: PLoS One. 2024 Feb 13;19(2):e0296834. doi: 10.1371/journal.pone.0296834 (PMC10863870; doi:10.1371/journal.pone.0296834)
Supplement: S4 Table — (PDF) [file pone.0296834.s006.pdf]

S4 Table. Baseline patient demographic and clinical characteristics of propensity score-matched intervention and control patients in the study (excluded prior to any complications existing).

Demographic Variables		Total		Intervention		Control	
		n	%	n	%	n	%
**Total**		81,726	100.00	27,242	100.00	54,484	100.00
**Sex**	**Male**	30,672	37.53	10,165	37.31	20,507	37.64
	**Female**	51,054	62.47	17,077	62.69	33,977	62.36
**Age**	**65–69**	34,275	41.94	11,240	41.26	23,035	42.28
	**70–74**	22,302	27.29	7,740	28.41	14,562	26.73
	**75–79**	13,681	16.74	4,612	16.93	9,069	16.65
	**80–84**	7,286	8.92	2,402	8.82	4,884	8.96
	**85–89**	3,247	3.97	984	3.61	2,263	4.15
	**90 +**	935	1.14	264	0.97	671	1.23
**Income quantile**	**1st quantile (poorest)**	16,322	19.97	5,522	20.27	10,800	19.82
	**2nd quantile**	10,623	13.00	3,508	12.88	7,115	13.06
	**3rd quantile**	12,706	15.55	4,240	15.56	8,466	15.54
	**4th quantile**	16,365	20.02	5,495	20.17	10,870	19.95
	**5th quantile**	25,710	31.46	8,477	31.12	17,233	31.63
**Findings or co-existing conditions at admission**	**History of hypertension**	53,790	65.82	18,078	66.36	35,712	65.55
	**History of diabetes mellitus**	7,972	9.75	2,621	9.62	5,351	9.82
	**History of hypertension and diabetes mellitus**	19,964	24.43	6,543	24.02	13,421	24.63
**Type of physician speciality**	**Internal medicine and family medicine**	71,473	87.45	23,805	87.38	47,668	87.49
	**Others**	10,253	12.55	3,437	12.62	6,816	12.51
**Type of public health insurance**	**National Health Insurance (self-employed)**	28,363	34.70	9,500	34.87	18,863	34.62
	**National Health Insurance (employees)**	53,156	65.04	17,675	64.88	35,481	65.12
	**Medical aid**	207	0.25	67	0.25	140	0.26
**PDC[1] > 1yr**	**< 290**	46,917	57.41	16,432	60.32	30,485	55.95
	**> = 290**	34,809	42.59	10,810	39.68	23,999	44.05
**PDC[1] > 2yr**	**< 290**	49,599	60.69	16,565	60.81	33,034	60.63
	**> = 290**	32,127	39.31	10,677	39.19	21,450	39.37

***$p < 0.001$, **$p < 0.05$, *$p < 0$.

[2]CRMHDP: Community-based Registration and Management for Hypertension and Type 2 Diabetes mellitus Project.

[1]PDC (proportion of days covered) calculation represents the number of total days covered divided by the number of total days in a given period.
