# Supplementary material for: Effect of motivated physicians and elderly patients with hypertension or type 2 diabetes mellitus in prepared communities on health behaviours and outcomes: A population-based PS matched retrospective cohort study during five-year follow-up period
Source: PLoS One. 2024 Feb 13;19(2):e0296834. doi: 10.1371/journal.pone.0296834 (PMC10863870; doi:10.1371/journal.pone.0296834)
Supplement: S5 Table — (PDF) [file pone.0296834.s007.pdf]

S5 Table. Difference-in-differences regression, including covariates of effects of the CRMHDP on the annual visits to clinics and annual days covered per person, with the control group matched by propensity scores.

Covariates		Annual visits to clinics		Annual days covered	
		Coeff.	S.E.	Coeff.	S.E.
Dependent variable	Intervention group (reference: control group)	-1.12 ***	0.045	-0.11	0.521
	Postintervention period (reference: preintervention period)	-0.30 ***	0.034	12.99 ***	0.436
	Group * Time	1.38 ***	0.063	26.29 ***	0.747
Covariates	Gender	0.31 ***	0.030	3.34 ***	0.372
	Age	-0.03 ***	0.002	-0.73 ***	0.029
	Income quartile	1.43 ***	0.050	-7.19 ***	0.610
	History of hypertension or type 2 DM	1.81 ***	0.034	-2.22 ***	0.428
	Type of physician speciality	-0.41 ***	0.042	0.98 *	0.520
	Type of Health Insurance	0.10 ***	0.030	-1.76 ***	0.371
	Presence of medical aid beneficiary	1.35 ***	0.229	-6.65 **	2.913
	Medication adherence 1 year before participation (Annual days covered)	0.01 ***	0.0002	0.66 ***	0.002
	Medication adherence 2 years before participation (Annual days covered)	0.002 ***	0.0002	0.03 ***	0.002
	Intercept	9.11 ***	0.175	117.75 ***	2.162
	Log Likelihood	-792862.4		-1609904	
	R-square	0.058		0.500	

***p < 0.01, **p < 0.05, *p < 0.1.
