# Supplementary material for: Effect of motivated physicians and elderly patients with hypertension or type 2 diabetes mellitus in prepared communities on health behaviours and outcomes: A population-based PS matched retrospective cohort study during five-year follow-up period
Source: PLoS One. 2024 Feb 13;19(2):e0296834. doi: 10.1371/journal.pone.0296834 (PMC10863870; doi:10.1371/journal.pone.0296834)
Supplement: Attachment — Submitted filename: Response to Reviewers.docx [file pone.0296834.s008.docx]

Dear an Editor,

**RE:** Authors’ responses to reviewers’ comments

**Title**: Effect of motivated physicians and elderly patients with hypertension or type 2 diabetes mellitus in prepared communities on health behaviours and outcomes: a population-based propensity score-matched retrospective cohort study over eight years

**Authors:** Eun Jee Park^1¶^, Hyunsung Kim^2¶^, Yaeji Lim^2^, Soon Young Lee^3*^, Weon-Young Lee^1*^

^1^ Department of Preventive Medicine, Chung-Ang University College of Medicine, 84 Heukseok-ro, Dongjak-gu, Seoul 06974, Republic of Korea

^2^ Department of Applied Statistics, Chung-Ang University, Seoul 03974, Republic of Korea

^3^ Department of Preventive Medicine and Public Health, Ajou University School of Medicine, Suwon-si 16499, Korea.

^¶^ These authors equally contributed to this work as the first authors

^*^ Corresponding authors

Weon-Young Lee, M.D. Ph.D.

**Journal of Plos One**

Please find point-by-point responses to the reviewers’ comments and an amended version of the manuscript with changes highlighted in red in the attached file. We are very grateful to the reviewers for their positive and useful suggestions, which have significantly improved the paper. We hope you will find the paper acceptable for your journal.

Thank you for your time. We look forward to hearing from you soon.

Yours sincerely,

Weon-young Lee

On behalf of all authors

Reviewer #1

This is a retrospective cohort using propensity score-paired matched control study of a Community based Registration and Management of Hypertension and Type 2 Diabetes mellitus Project (CRMHDP) that applying financial incentives activating both primary care physicians/patients and public health promotion team resulted in a statistically significant difference for type 2 DM patients for short and long term health outcomes in certain cities of Korea.

Among the countries applying system of universal coverage healthcare, I appreciated the hard work and determination of experimentally delivering such a high quality of healthcare for the personalized control of type 2 DM in Korea. It is well designed in various domains of healthcare system including incentives, followup strategy and additional community support for the health promotion. This an excellent longterm healthcare example of type 2 DM for other regions and other countries as well.

Comments:

Q1. The control group defined as the patient’s clinical characteristics (presence of hypertension or/and type 2 DM). It's better to exclude the patients with hypertension to have pure control group of type 2 DM.

Answer) Participants in this study were selected as intervention subjects who were diagnosed with hypertension, type 2 DM or/and hypertension and type 2 DM. Basically, we matched the control group as in the case above. As shown in table S1 and S2, it was statistically confirmed that there was no difference in the distribution of hypertension or/and type 2 DM in each group.

Q2. The parameters of long term outcome could consider either the frequency of emergent service of the target patients experienced or their annual/total medical expenditures during the followup years to disclose the severity and long-term financial burden of disease.

Answer) Right. As you mentioned on the comments above, it is important to evaluate the long term financial burden of disease such as frequency of emergent service or annual/total medical expenditure experienced by patients. In this study, hospital admission due to major complications in hypertension or type 2 DM is also meant to be admission from emergent service of the target patients experienced. We stated the inclusion of emergent service admission on the outcome variable section. Also, the economic evaluation is important as part of the research area on this study. Further study for the economical evaluation (includes total annual medical expenditure) would definitely be needed. In our study, instead, we stated that further study should be required in this area in the conclusion section. Modified text was marked by blue collar

“… Moreover, further study for economical evaluation including total medical expenditure would be needed in order to disclose the severity and long-term financial burden of the diseases.” (conclusion section)

Q3. The parameters using in the propensity score-matched pair method could consider other pre-existed medical co-morbidities besides hypertension and type 2 DM, such as COPD, hepatic and renal diseases, etc.

Answer) As you mentioned, we couldn’t consider pre-existed co-morbidities besides hypertension and type 2 DM like COPD, hepatic and renal diseases, etc. However, patients diagnosed with catastrophic disease such as cancer, stroke, and myocardial infarction, rare and intractable disease were excluded to avoid confounding bias due to the differences in the presence of catrastrophic disease between the intervention and control group. This would be limitation of our study. We also stated that on the discussion section. Modified text was marked by blue collar

“… Moreover, we could not consider pre-existed co-morbidities besides hypertension and type 2 DM like COPD, hepatic and renal diseases, etc. However, patients diagnosed with catastrophic disease such as cancer, stroke, and myocardial infarction, rare and intractable disease were excluded to avoid confounding bias due to the differences in the presence of catastrophic disease between intervention and control group. ….” (discuss section)

Q4. The format of tables and figures needs to be revised as journal requested.

Answer) Yes, we revised those tables and figures as journal requested.

Reviewer #2: In this manuscript, Park et al. present findings indicating that patients with effectively managed hypertension and/or DM have better outcomes. Overall, the paper demonstrates good writing quality and grammatical accuracy. The results are clear and straightforward. However, there are concerns regarding the reported results that need to be addressed before considering the manuscript for publication.

Results:

Q1. Figure 1: After excluding patients with disease-related complications and applying 1:2 matching, the numbers in the intervention group and control group are 27865 and 93730, respectively. Should these numbers be 27242 and 54484, respectively?

Answer) It was rewritten to correct errors. Modified text was marked by blue collar

Q2. Table 2: Hazard ratios were calculated based on the intervention group (n = 46865) versus the control group (n = 93730). It is important to note that myocardial infarction and stroke may have a tendency for recurrence in certain cohorts. Could you provide hazard ratios based on the intervention group (n = 27242) versus the control group (n = 54484), which excludes the pre-existing complications? Additionally, it would be valuable to include data on the outcomes including admission, emergency department visits, or hospital stays during the follow-up period when comparing these two groups.

Answer) Results of Table 2 is for the HR which excludes the pre-existing complications. We evidently re-mentioned on the title of the Table 2. Modified text was marked by blue collar.
