# Supplementary material for: Effect of motivated physicians and elderly patients with hypertension or type 2 diabetes mellitus in prepared communities on health behaviours and outcomes: A population-based PS matched retrospective cohort study during five-year follow-up period
Source: PLoS One. 2024 Feb 13;19(2):e0296834. doi: 10.1371/journal.pone.0296834 (PMC10863870; doi:10.1371/journal.pone.0296834)
Supplement: Attachment — Submitted filename: Response to Reviewers_0927.docx [file pone.0296834.s009.docx]

Dear an Editor,

**RE:** Authors’ responses to reviewers’ comments

**Title**: Effect of motivated physicians and elderly patients with hypertension or type 2 diabetes mellitus in prepared communities on health behaviours and outcomes: a population-based propensity score-matched retrospective cohort study over eight years

**Authors:** Eun Jee Park^1¶^, Hyunsung Kim^2¶^, Yaeji Lim^2^, Soon Young Lee^3*^, Weon-Young Lee^1*^

^1^ Department of Preventive Medicine, Chung-Ang University College of Medicine, 84 Heukseok-ro, Dongjak-gu, Seoul 06974, Republic of Korea

^2^ Department of Applied Statistics, Chung-Ang University, Seoul 03974, Republic of Korea

^3^ Department of Preventive Medicine and Public Health, Ajou University School of Medicine, Suwon-si 16499, Korea.

^¶^ These authors equally contributed to this work as the first authors

^*^ Corresponding authors

Weon-Young Lee, M.D. Ph.D.

**Journal of Plos One**

Please find point-by-point responses to the reviewers’ comments and an amended version of the manuscript with changes highlighted in red in the attached file. We are very grateful to the reviewers for their positive and useful suggestions, which have significantly improved the paper. We hope you will find the paper acceptable for your journal.

Thank you for your time. We look forward to hearing from you soon.

Yours sincerely,

Weon-young Lee

On behalf of all authors

Reviewer #3

Comments:

Q1. The authors should add other pre-existed medical co-morbidities such as COPD, hepatic and renal diseases, etc. into the propensity score-matched pair method. For pre-existed medical co-morbidities, the authors can identify based on ICD codes from the NHIS database.

Answer) As you mentioned, we couldn’t consider pre-existed co-morbidities besides hypertension and type 2 DM like COPD, hepatic and renal diseases, etc. However, patients diagnosed with catastrophic disease such as cancer, stroke, and myocardial infarction, rare and intractable disease were excluded to avoid confounding bias due to the differences in the presence of catastrophic disease between the intervention and control group. This would be limitation of our study. We also stated that on the discussion section. Modified text was marked by blue collar

“… Moreover, we could not consider pre-existed co-morbidities besides hypertension and type 2 DM like COPD, hepatic and renal diseases, etc. However, patients diagnosed with catastrophic disease such as cancer, stroke, and myocardial infarction, rare and intractable disease were excluded to avoid confounding bias due to the differences in the presence of catastrophic disease between intervention and control group. ….” (discuss section)

Q2. 2. P6. The authors mentioned the study follow-up period (2010/11–2015/16).

P5. They also mentioned that 2010–2011 was used as the baseline years, while the follow-up period was until 2015–2016. P3. This study aimed to examine the impact of the CRMHDP on

healthcare use and the health outcomes of patients using a population-based propensity score-matched retrospective cohort study over “eight” years. These time periods are not consistent. Please specify exactly the start year and the end year of the follow-up. Most importantly, when is the implementation period for CRMHDP? To assess the effect of CRMHDP, the suitable time period should be before and after the implementation period for CRMHDP.

Answer) We made a mistake as writing the title words of this paper. As you mentioned, we replaced ‘over eight years’ with during five-year follow-up period in the title of the manuscript. This study used the past medication rates in two-year pre-intervention period as an confounding factor examing the effect of CRMHDP on the process and health outcome of health care service, which means that this study considers before and after the implementation period for CRMHDP. Modified text was marked by blue collar

“The clinical variables were the presence of the coexistence of hypertension or type 2 DM, the past medication adherence prior to participation in the study, and the type of speciality by the physician treating the patients in the study. The past medication adherence rates during the two-year per-intervention period, were measured by the PDC. (Method section)”

3. For health outcomes, it would be valuable to include data on the outcomes including admission, emergency department visits, or hospital stays during the follow-up period when comparing these two groups.

Answer) In this study, we used total mortality and hospital admission due to major complications in hypertension or type 2 DM as outcome variables. However, emergency department visits and hospital stays were not employed. It was included as an limitation of this study.

“… Lastly, this study did not included emergency room visits and hospital stays as another health outcome ..” (discussion section)

4. The authors can perform a sub-group analysis according to Hypertension and diabetes. Since the CRMHDP covers HTN and DM, it is essential to examine the effect separately.

Answer) We did not examine the effect of the CRMHDP on hypertension and type 2 DM separately due to the following reasons. According to the existing literature^[1],^^[2]^, various type of incentives including financial support to patient and physician can lead to improve the health outcome (BP or blood glucose control) or medication adherence of hypertension and type 2 DM. This study assumed the CRMHDP would have worked type 2 DM as well as hypertension simultaneously. Moreover, type 2 DM and essential hypertension are common conditions that are frequently present together. Patients with type 2 DM combining hypertension accounted for about 25% of the entire subject of this study. However, since our assumption of the effect of the CRMHDP on them may be wrong, the results of this study should be interpreted very cautiously. We added this text to the discussion section as a limitation of this study.

5. The format of tables and figures needs to be revised as journal requested.

Answer) Yes, we revised those tables and figures as journal requested.

Reviewer #4: The authors present a manuscript investigating the effectiveness of chronic disease management for elderly patients with hypertension and/or Type 2 Diabetes mellitus. Using a population-based propensity score-matched retrospective cohort study, health outcomes and utilisation of healthcare in four cities in South Korea were investigated with a focus of estimation the effect of motivated physicians, patients and community support on these outcomes.

The manuscript has undergone a first revision.

The work per se is interesting and important but the statistical analysis and description is lacking information.

1.) First of all there should be some sample size considerations justifying why they included patients from 16 months‘ project (CRMHDP) time.

Answer) We made change of the relevant text in stud design and sample of method section as follows.

“ the CRMHDP group (n = 48,325), designated as the intervention group, was composed of patients who enrolled in the project via participating local clinics (n = 409) which accounted for 84.2% of all local clinics (n = 486), in the cities, during the baseline period (September 1, 2010, to December 31, 2011). The local clinics not engaged in the project had few patients with hypertension and type 2 DM because their clinical practitioners were specialist such as opthamologist, pediatric physician, otolaryngologist and opthalmologis. Therefore, the registered patients can represent all elderly hypertensive and diabetic patient in the cities.”

2.) The authors state that they have excluded patients with complications during the baseline year – what about death?

Answer ) We excluded died patient from this study. It was added to exclusion criteria in the method section

3.) In the section „study design and sample“ they state that the baseline period is September 1, 2010 to December 31, 2011. This is incorrectly stated in Fig. 1 – 2010 to 2010 and it is also misleading in the text where they write in the first sentence in the section „study design and sample“ 2010/2011 are used as baseline years. Why did not they start with the baseline period on January 1, 2010?

Answer) We corrected the misspelling in Figure 1(2010). Registration to the project was recommended a elderly patient with hypertension and type 2 DM aged 65 and over by a physician at local clinic. It took one and half year for 48,325 subjects of the project to were registered in local clinics.

4.) The eight year coverage is also not clearly explained. If a patient is included in Sept 1, 2010 his/her observation period would be Sept 1, 2008 to Sept 1, 2015 and if a patient is included at the end of 2011 his/her observation period would be end of 2009 to end of 2016 – for me this are seven instead of 8 years (see also statistical analysis - during a period of two years prior to and five years after initial registration).

Answer) We made a mistake as writing the title words of this paper. As you mentioned, we replaced ‘over eight years’ with during five-year follow-up period in the title of the manuscript.

5.) As for propensity score matching the compliance of antihypertensive and antidiabetic drugs type DM during the two years prior to baseline years is included. How was this considered regarding inclusion/exclusion of patients. What is known about the duration of the diseases – how is this incorporated?

Answer) This study used the past medication rates in two-year pre-intervention period as an confounding factor examing the effect of CRMHDP on the process and health outcome of health care service, which means that this study considers before and after the implementation period for CRMHDP. However, we did not consider the duration of the disease because it could not be identified in the KNHIS (Korean National Health Insurance) data. We included it as an study limitation of the discussion section. Modified text was marked by blue collar.

6.) They describe in a section the propensity score calculation and matching and then they have a section on independent variables – redundant?

Answer) The section the propensity score calculation addressed independent variables as matching one whereas the section independent variable explained how to measure them. We think that to keep it would be helpful for journal readers

7.) To show the quality of matching it would be good not only to have supplementary tables but to include a figure as e.g. in Riedl et al. Plos One 2016.

Answer) As one of the methods of evaluating the quality of matching, our study presented the standardized difference in measures (Table S1, Table S2), confirming that the difference between the intervention and control group was less than 10%. As a result, we have suggested through the result in the tables that the matching has been properly balanced. We also show the additional box plot to show the quality of matching properly (see figure 4).

8.) How did the authors handle matching of patients in their statistical analysis – this information has to be included in the statistical analysis section.

Answer) The description of the matching is explained in detail in the ‘Propensity score calculation and matching’ section (manuscript page 6).

9.) PS matching should result in two similar groups – therefore I would also like to see non-adjusted results beside the adjusted results.

Answer) Table S1, Table S2 contain statistical result and statistical test for variables before and after matching (see supplement Table S1 and Table S2).

10.) What about missing data and how were they handled?

Answer) If there is missing in the variable or there is no control patient within the proper range of the propensity score, these data are eliminated all. Those data are not included in the neither intervention nor control group in this study. Therefore, there is no missing in the matching variables of the data used in the analysis.

11.) In the result section the interpretation has to be checked – increased, decreased seems to be confused sometimes and also the time period is not clear - one year before and after participation (preintervention period – postintervention period) – see table 1 for instance.

Answer) We modified the sentence as written below. Modified text was marked by blue collar in the manuscript.

**This implied that the intervention increased the annual clinic visits per person one year before and after the intervention.**

-> “This implied that the intervention contributed to escalate the annual clinic visits per person one year before and a year after the intervention.” (Results section, page 8)

**This meant that the intervention led to an increase in the annual days covered for antihypertensive, antidiabetic drug and insulin injections.**

-> “This meant that the intervention led to strengthen compliance in the annual days covered for antihypertensive, antidiabetic drug and insulin injections.” (Results section, page 8)

12.) More relevant literature from European countries should be included!

Answer) We included some more relevant European literature in the text. Modified text was marked by blue collar in reference list. (Reference section)

1. Hulbert LR, Michael SL, Charter-Harris J, Atkins C, Skeete RA, Cannon MJ. Effectiveness of Incentives for Improving Diabetes-Related Health Indicators in Chronic Disease Lifestyle Modification Programs: a Systematic Review and Meta-Analysis. Prev Chronic Dis. 2022 Oct 27;19:E66. doi: 10.5888/pcd19.220151. PMID: 36302383; PMCID: PMC9616129.
2. Kong E, Beshears J, Laibson D, Madrian B, Volpp K, Loewenstein G, Kolstad J, Choi JJ. Do physician incentives increase patient medication adherence? Health Serv Res. 2020 Aug;55(4):503-511. doi: 10.1111/1475-6773.13322. PMID: 32700389; PMCID: PMC7376007.
