# Supplementary material for: Effect of motivated physicians and elderly patients with hypertension or type 2 diabetes mellitus in prepared communities on health behaviours and outcomes: A population-based PS matched retrospective cohort study during five-year follow-up period
Source: PLoS One. 2024 Feb 13;19(2):e0296834. doi: 10.1371/journal.pone.0296834 (PMC10863870; doi:10.1371/journal.pone.0296834)
Supplement: Attachment — Submitted filename: Response to Reviewers_Second revision_1113.docx [file pone.0296834.s010.docx]

Dear an Editor,

**RE:** Authors’ responses to reviewers’ comments

Effect of motivated physicians and elderly patients with hypertension or type 2 diabetes mellitus in prepared communities on health behaviours and outcomes: a population-based PS matched retrospective cohort study during five-year follow-up period

Eun Jee Park^1¶^, Hyunsung Kim^2¶^, Yaeji Lim^2^, Soon Young Lee^3*^, Weon-Young Lee^1*^

^1^ Department of Preventive Medicine, Chung-Ang University College of Medicine, 84 Heukseok-ro, Dongjak-gu, Seoul 06974, Republic of Korea

^2^ Department of Applied Statistics, Chung-Ang University, Seoul 03974, Republic of Korea

^3^ Department of Preventive Medicine and Public Health, Ajou University School of Medicine, Suwon-si 16499, Korea.

^*^ Corresponding authors:

Weon-Young Lee, M.D. Ph.D.

Department of Preventive Medicine, Chung-Ang University College of Medicine, 84 Heukseok-ro, Dongjak-gu, Seoul 03974, Republic of Korea

Tel +82-2-820-5695; e-mail: [wylee@cau.ac.kr](mailto:wylee@cau.ac.kr)

Soon Young Lee, M.D. Ph.D.

Department of Preventive Medicine and Public Health, Ajou University School of Medicine, Suwon-si 16499, Korea.

Tel: +82-031-219-5012; e-mail: [solee5301@gmail.com](mailto:solee5301@gmail.com)

^¶^ These authors equally contributed to this work as the first authors

Editors comment

As requested by National Health Insurance Service (NHIS), the data provider, the research number below is listed on the Manuscript.

Acknowledgement: This study used NHIS-NSC data (NHIS-2020-1-178) from the National Health Insurance Service (NHIS).

Reviewer #3

Comments:

Q1. For using administrative database, it is weird that the authors have difficulties to incorporate other pre-existing comorbidities. Except this, however, the authors have not adequately addressed my comments regarding a sub-group analysis according to Hypertension and diabetes. The authors mentioned only 1/4 of individuals with both HTN and DM. Hence, it is adequate to perform the sub-group analysis to examine the effect in more details.

Answer) We can understand your concerns regarding to not making a sub-group analysis according to Hypertension and Diabetes. However, according to the huge existing literature (36-38) related to health behavioural economics, financial incentives to chronic patient and physician for the change of patient behavior has been very effective tool at changing behaviors in chronic condition related to medication adherence. Hence, this study assumed the CRMHDP would have positive impact on patient behavior including medication adherence among both hypertensive patients and diabetic patients. However, since our assumption of the CRMHDP’s impact on them may be wrong, a sub-group analysis should be made in the further. We changed the text of discussion section as mentioned above.

Added the citation also in the reference list

36) Hohmann NS, Hastings TJ, Jeminiwa RN, Qian J, Hansen RA, Ngorsuraches S, Garza KB. Patient preferences for medication adherence financial incentive structures: A discrete choice experiment. Res Social Adm Pharm. 2021 Oct;17(10):1800-1809. doi: 10.1016/j.sapharm.2021.01.018. Epub 2021 Feb 5. PMID: 33608244; PMCID: PMC9070307.

37) Adams AS, Madden JM, Zhang F, Soumerai SB, Gilden D, Griggs J, Trinacty CM, Bishop C, Ross-Degnan D. Changes in use of lipid-lowering medications among black and white dual enrollees with diabetes transitioning from Medicaid to Medicare Part D drug coverage. Med Care. 2014 Aug;52(8):695-703. doi:

38) Kong E, Beshears J, Laibson D, Madrian B, Volpp K, Loewenstein G, Kolstad J, Choi JJ. Do physician incentives increase patient medication adherence? Health Serv Res. 2020 Aug;55(4):503-511. doi: 10.1111/1475-6773.13322. PMID: 32700389; PMCID: PMC7376007.

Reviewer #4: The answers to most of my questions were appropriate.

Q1. The answer to the question how the authors handled matching of patients in their statistical analysis was not adequate. The matching and the ratio of 1:2 has to be considered in the statistical analysis section (use the appropriate variance estimator) and not in the matching process (Propensity score calculation and matching’ section).

Answer) We included the text about the matching and the ratio of 1:2 in the statistical analysis section, as you mentioned above (page 6).

The introduction is as follows,

“ The analysis was based on propensity score matching(PSM) which was first proposed by Rosenbaum and Rubin in the 1980 (17) ~”

Added the citation also in the reference list

(17) Rosenbaum PR, Rubin DBJB. The central role of the propensity score in observational studies for causal effects. 1983;70(1):41-55.

Q2. Concerning missing data: it should be made clear what they have done- that means they should include their answer in the text. "" If there is missing in the variable or there is no control patient within the proper range of the propensity score, these data are eliminated all. Those data are not included in the neither intervention nor control group in this study. Therefore, there is no missing in the matching variables of the data used in the analysis."

Answer ) We included our answer about missing data evidently in the statistical analysis section. (page 7, 9-11 lines)

"If there is missing in the variable or there is no control patient within the proper range of the propensity score, these data are eliminated all. Those data are not included in the neither intervention nor control group in this study. Therefore, there is no missing in the matching variables of the data used in the analysis. "

Q3. The authors have to check the references - the journal names are not included?

Answer ) The references are organized in Vancouver style using End Note program according to the journal’s reference style requirement. The additional literature has also been rearranged in the same.
